# *Clinacanthus nutans* Extracts Are Antioxidant with Antiproliferative Effect on Cultured Human Cancer Cell Lines

**DOI:** 10.1155/2013/462751

**Published:** 2013-02-27

**Authors:** Yoke Keong Yong, Jun Jie Tan, Soek Sin Teh, Siau Hui Mah, Gwendoline Cheng Lian Ee, Hoe Siong Chiong, Zuraini Ahmad

**Affiliations:** ^1^Department of Biomedical Science, Faculty of Medicine and Health Sciences, Universiti Putra Malaysia (UPM), 43400 UPM Serdang, Malaysia; ^2^Advanced Medical and Dental Institute, Universiti Sains Malaysia, 13200 Kepala Batas, Penang, Malaysia; ^3^Department of Chemistry, Faculty of Science, Universiti Putra Malaysia (UPM), 43400 UPM Serdang, Malaysia

## Abstract

*Clinacanthus nutans* Lindau leaves (CN) have been used in traditional medicine but the therapeutic potential has not been explored for cancer prevention and treatment. Current study aimed to evaluate the antioxidant and antiproliferative effects of CN, extracted in chloroform, methanol, and water, on cancer cell lines. Antioxidant properties of CN were evaluated using DPPH, galvinoxyl, nitric oxide, and hydrogen peroxide based radical scavenging assays, whereas the tumoricidal effect was tested on HepG2, IMR32, NCL-H23, SNU-1, Hela, LS-174T, K562, Raji, and IMR32 cancer cells using MTT assay. Our data showed that CN in chloroform extract was a good antioxidant against DPPH and galvinoxyl radicals, but less effective in negating nitric oxide and hydrogen peroxide radicals. Chloroform extract exerted the highest antiproliferative effect on K-562 (91.28 ± 0.03%) and Raji cell lines (88.97 ± 1.07%) at 100 **μ**g/ml and the other five cancer cell lines in a concentration-dependent manner, but not on IMR-32 cells. Fourteen known compounds were identified in chloroform extract, which was analysed by gas chromatography—mass spectra analysis. In conclusion, CN extracts possess antioxidant and antiproliferative properties against cultured cancer cell lines, suggesting an alternate adjunctive regimen for cancer prevention or treatment.

## 1. Introduction

Cancer remains one of the major health threats to Malaysian population. Statistics have shown that the annual mortality rate of cancer patients in hospitals of Malaysian Ministry of Health has consistently reached 10-11% since 2006 [1]. Increasing evidence has demonstrated that tumorigenesis is closely associated with elevated level of intracellular free radicals, the reactive oxygen/nitrogen species (RONS), which trigger cancer initiation and progression [2–4]. RONS are commonly generated as metabolic by-products in normal cells and have an indispensable role in redox-mediated signaling. However, RONS level needs to be maintained through a redox homeostasis to keep the basal RONS below cytotoxic level. Uncontrolled RONS production overwhelming the endogenous antioxidant capacity will result in detrimental damage to cellular protein, lipid, and DNA, leading to genomic instability, and ultimately promotes cancer formation [5–7]. Evidence has shown that oncogene activation, increased metabolic activity, and mitochondrial malfunction are common causes which are responsible for substantial surge in cellular RONS level [8]. Therefore, scavenging RONS with antioxidant supplement could salvage cells from oxidative stress and prevent cancer growth and expansion. This benefit had also been seen in patients at risk of breast cancer, who were saved from the disease by taking antioxidant supplement, such as carotenoids [9]. With advances in cancer research, many molecular targeted drugs have been introduced and showed promising outcome with little side effects. However the use has also been limited by genomic instability and drug resistance characteristics in certain cancer cells. Thus, interest has been shifted to study traditional herbs as alternate anticancer regimens due to its multitargeted characteristics. Hence, we sought to evaluate *Clinacanthus nutans* Lindau for its potential to be used as natural nutraceuticals for cancer prevention and treatment. *Clinacanthus nutans* Lindau (CN), also known as Sabah Snake Grass, from the family Acanthaceae, is a type of famous medicinal plants in Thai folklore medicine. Previously, CN has traditionally been used to treat inflammation [10] and viral infection [11, 12]. The use of this herb has also been translated in clinics to treat herpes infection in Thailand [13]. The cholophyll derivatives (phaeophytins) of CN chloroform extracts contained 13^2^-hydroxy-(13^2^-*R*)-phaeophytin b, 13^2^-hydroxy-(13^2^-*S*)-phaeophytin a, and 13^2^-hydroxy-(13^2^-*R*)-phaeophytin and exhibited antiherpes simplex activity [14], potentially through herpes virus inactivation and inhibition preinfection [15]. Despite all the known biological activities from previous work, emerging lay testimonies and Malaysian newspaper reports suggested that CN possesses antitumor effects and had saved many of various cancers. However, these testimonies were not supported by scientific evidence. We hypothesize that CN derivatives could be a source of cytoprotective antioxidant based anticancer regimen. Hence, the main objective in this study was to examine the antioxidant and cytotoxicity effects of *C. nutans* on cancer cell lines.

## 2. Methods and Materials

### 2.1. Chemicals

1,1-diphenyl-2-picrylhydrazyl (DPPH) radical, galvinoxyl radical, 6-hydroxy-2,5,7,8-tetramethylchroman-2-carboxylic acid (Trolox), phosphate buffer saline (PBS), trypan blue, 3-(4,5-Dimethylthiazol-2-yl)-2,5-diphenyltetrazolium bromide (MTT), and 10x trypsin-EDTA solution were purchased from Sigma Chemical Co. Ltd., Malaysia. Hydrogen peroxide and sodium nitroprusside (SNP) were purchased from Merck, Malaysia. All the cancer cell lines, including human liver hepatocellular carcinoma (HepG2), human neuroblastoma cell line (IMR-32), human lung cancer cell line (NCI-H23), human gastric cancer cell line (SNU-1), human colon adenocarcinoma cell line (LS-174T), human erythroleukemia cell line (K-562), human cervical cancer cell line (HeLa), and human Burkitt's lymphoma cell line (Raji), were purchased from the American Type Culture Collection (ATCC, Rockville, MD), while human umbilical veins endothelial cells (HUVECs) were obtained from Cascade Biologics, Inc. (Portland).

### 2.2. Plant Materials

Whole plant of *Clinacanthus nutans* (Burm.f.) Lindau was harvested in November 2011 from botanical garden in Serdang, Selangor, Malaysia. The botanical identify of *C. nutans* was characterized by the Phytomedicinal Herbarium, Institute of Bioscience, Universiti Putra Malaysia, Selangor (Voucher no. SK1980/11).

### 2.3. Preparation of Extracts

The *C. nutans* leaves were freshly harvested and oven-dried at 40–45°C. The leaves were then ground into powder and sequentially soaked in chloroform (CNC), methanol (CNM), and distilled water (CNA) at room temperature. The extracts in all three solvents were collected separately in clean glass bottles. Extracts were then filtered using Whatman no. 1 paper. The filtrates in chloroform and methanol were dried by using rotary evaporator, while filtrates in distilled water were dried with a freeze drier. All the extracts were kept at 20°C until use.

### 2.4. Determination of Antioxidant Properties

#### 2.4.1. DPPH Radical Scavenging Assay

DPPH radical scavenging activity of the tested extracts was determined according to the method described by Chan et al. [16] with slight modifications. In brief, each extract solution (50 *μ*L), after serially diluted in ethanol to 12.5, 25, 50, and 100 *μ*g/mL, was mixed with 195 *μ*L of DPPH ethanolic solution (0.2 mM). Then, the mixtures were swirled gently for 1 min and kept in dark for 60 min. The DPPH radical scavenging activity of each extract was determined by electron spin resonance spectrometry (JEOL-JES-FA100, Tokyo, Japan) using the following parameters: sweeping field, 340.047 ± 5 mT; microwave power, 4 mW; modulation width, 0.1 mT; sweep time, 30 s; time constant, 0.1 s; and amplitude, 160. Trolox was used as the standard and DPPH radical scavenging activity of each extract was expressed as *μ*g Trolox equivalent/g extract.

#### 2.4.2. Galvinoxyl Radical Scavenging Activity

In order to determine the galvinoxyl radical scavenging activity of each extract. Extracts were serially diluted to 12.5, 25, 50, and 100 *μ*g/mL solutions in ethanol, and each of them (50 *μ*L) was mixed with 150 *μ*L of galvinoxyl radical ethanolic solution (0.125 mM). Then, the mixture was swirled gently for 1 min and kept in the dark for 60 min. Galvinoxyl radical scavenging activity of each extract was determined by electron spin resonance spectrometry (JEOL-JES-FA100, Tokyo, Japan) using the following parameters: sweeping field, 340.047 ± 5 mT; microwave power, 4 mW; modulation width, 0.2 mT; sweep time, 30 s; time constant, 0.1 s; and amplitude, 120. Trolox was used as the standard and galvinoxyl radical scavenging activity of each extract was expressed as *μ*g Trolox equivalent/g extract.

#### 2.4.3. Nitric Oxide (NO) Radical Scavenging Assay

Sodium nitroprusside (SNP) spontaneously generated nitric oxide (NO) by interacting with oxygen in aqueous solution at physiological pH (pH 7.4). Scavenger of NO will eventually reduce the production of nitrite ion in solution which can be determined by the use of Griess reagent. Briefly, each extract (50 *μ*L) of various concentrations (12.5, 25, 50, and 100 *μ*g/mL) was mixed with equal volume of SNP (10 mM final concentration), in phosphate buffer saline (PBS) and incubated at room temperature for 150 min [17]. DMSO in distilled water (0.1%) without the extract served as the negative control and quercetin was used as a positive control. After incubation, the samples from the above were reacted with equal volume of Griess reagent (1% sulphanilamide, 0.1% naphthylethylenediamine dichloride, and 3% phosphoric acid). The absorbance of the chromophore which was formed in the reaction was measured at 550 nm against the corresponding control. The amount of nitrite in the samples was calculated from sodium nitrite standard curve (0–100 *μ*M).

#### 2.4.4. Scavenging Activity of Hydrogen Peroxide

All three extracts of *C. nutans* leaves were tested for scavenging potential against hydrogen peroxide according to the Ruch et al. [18]. Briefly, sample extract (2 mL) with different concentration (12.5, 25, 50, and 100 *μ*g/mL) was mixed with hydrogen peroxide (H_2_O_2_) solution (1.2 mL, 40 mM) in phosphate buffer (pH 7.4). The mixture was incubated for 10 min, and the absorbance was measured at 230 nm. An equal volume of distilled water without H_2_O_2_ served as blank. The scavenging activity of each extract was calculated by using the formula as follows: % scavenging activity = [(*A*_*c*_ − *A*_*t*_)/*A*_*c*_] × 100, where *A*_*c*_ indicates the absorbance of the control and *A*_*t*_ represents the absorbance of the extract.

### 2.5. Cell Culture

Three different extracts were tested on eight human cancer cell lines, including human liver hepatocellular carcinoma (HepG2), human neuroblastoma cell line (IMR-32), human lung cancer cell line (NCI-H23), human gastric cancer cell line (SNU-1), human colon adenocarcinoma cell line (LS-174T), human erythroleukemia cell line (K-562), human cervical cancer cell line (HeLa), human Burkitt's lymphoma cell line (Raji) and normal cell, and human umbilical veins endothelial cells (HUVECs) for their antiproliferative activity. All the cell lines were maintained in RPMI 1640 or DMF medium containing 10% (v/v) fetal bovine serum (FBS) supplemented with penicillin (100 U/mL) and streptomycin (100 *μ*g/mL), except HUVECs which were grown in M200 supplemented with low serum growth supplement at 37°C under humidified atmosphere containing 5% CO_2_ in the incubator.

### 2.6. Antiproliferation Assay

Antiproliferative activity of the three extracts was examined by using MTT assay [19]. Briefly, cells (6 × 10^3^ cells/mL) were seeded in 96-well, flat bottomed plate and treated with three different extracts at a concentration range from 3.125 to 100 *μ*g/mL after cells were incubated at 37°C in a humidified 5% CO_2_/95% air mixture for overnight. After 72 h of posttreatment, 20 *μ*L of 0.5% 3(4,5-dimethyl-thiazol-2-yl)2,5-diphenyltetrazolium bromide (MTT) solution in phosphate buffer saline (PBS) was added to each well and incubated for additional 4 h in 5% CO_2_ humidified incubator. The plate was centrifuged at 3000 rpm for 10 min and the supernatant was removed. The formazan crystals in each well were dissolved by adding 100 *μ*L of dimethyl sulfoxide (DMSO). The amount of purple formazan produced relative to number of viable cells was determined by measuring the absorbance with a microplate spectrophotometer reader at 550 nm. For CN treated cells, viability was expressed as a percentage of control cells. All the assays were carried out in triplicate and in three independent tests. All the extracts were dissolved in DMSO at final concentration less than 0.1%. Under these conditions, DMSO was not toxic to all the cancer cell lines mentioned above.

### 2.7. GC-MS Analysis

CNC was analyzed by gas chromatography equipped with mass spectrometry (GC-MS-QP2010 Plus-Shimadzu). The column temperature was set to 50°C for 4 min, then increased to 320°C at the rate of 7°C/min, and then held for 20 min. The injector temperature was set at 280°C (split mode with the ratio being adjusted to 20 : 1, injection volume = 0.1 *μ*L). The flow rate of the helium carrier gas was set to 1 mL/min. Total run time was 60 min. Mass spectra were obtained from the range *m/e* 40 to 700 and the electron ionization at 70 eV. The chromatograms of the sample were identified by comparing their mass spectra with NIST08 library data, and the GC retention time against known standards.

### 2.8. Statistical Analysis

All the data presented in mean ± standard error of mean (SEM) and performed by using the Statistical Package for Social Sciences (SPSS 16). Data were analyzed by one-way ANOVA, followed by Dunnett's test. *P* < 0.05 was considered to be significant.

## 3. Results

### 3.1. DPPH Radicals Scavenging Activity

**Figure 1:**
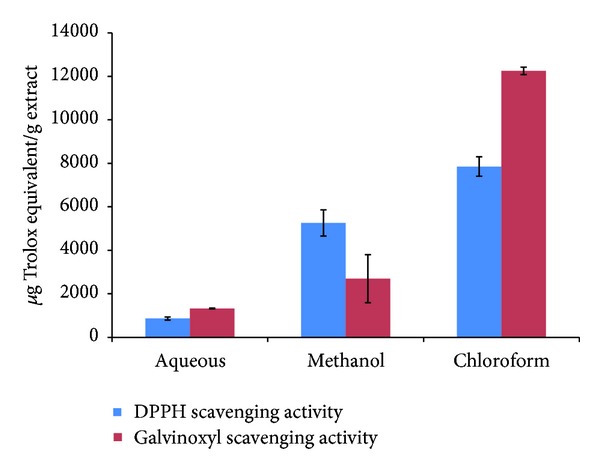
DPPH and galvinoxyl radical scavenging activity of various solvent extracts of *Clinacanthus nutans* leaves. Results represent mean ± SEM (*n* = 3).

DPPH has been widely used to measure the antioxidant property of various samples including fruits, beverages, and even plant extracts. In the current study, Trolox was used as standard, and hence the antioxidant capacity of an extract will be expressed in *μ*g Teq/g extract.  Figure 1 illustrates that the CNC was found to exhibit the highest DPPH scavenging activity compared to CNM and CNA with an antioxidant capacity value, 7852.63 ± 449.90 *μ*g Teq/g extract. However, CNA showed the lowest activity with antioxidant capacity value 864.11 ± 73.49 *μ*g Teq/g extract. The antioxidant activity of the *C. nutans* extracts decreased in the order of chloroform > methanol > aqueous.

### 3.2. Galvinoxyl Scavenging Activity

Galvinoxyl radical scavenging activity of serially extraction of *C. nutans* was evaluated as the same manner of DPPH where the Trolox was used as standard and the absorbance was measured by using ESR spectra.  Figure 1 showed the galvinoxyl radical scavenging activity of three types of extractions. Chloroform extract possessed the highest scavenging activity with the value of 12248.82 ± 173.50 *μ*g Teq/g extract. Moreover, a similar order was observed where the order of galvinoxyl radical scavenging activity was chloroform > methanol > aqueous.

### 3.3. Nitric Oxide Scavenging Activity

**Figure 2:**
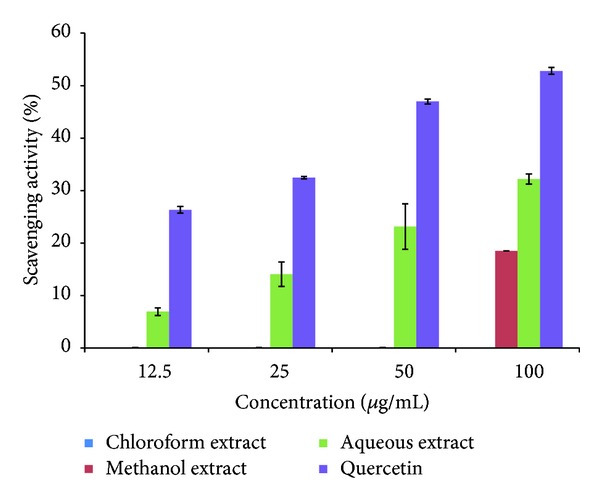
Percentage scavenging activity of nitric oxide radicals in response to *C. nutans* extracts in different solvents at different concentrations. The reaction was performed in triplicates and results were expressed in % inhibition (mean ± SEM).

Figure 2 showed the nitric oxide (NO) scavenging activity of three different extracts of *C. nutans* leaves. It was observed that only CNA has the ability to scavenge nitric oxide radical and was in the concentration dependent manner. The highest NO scavenging activity of 32.33 ± 0.97% was observed using 100 *μ*g/mL of CNA. Nitrite content was significantly higher in CNM at 100 *μ*g/mL with NO scavenging activity of 18.51 ± 8.24%.

### 3.4. Hydrogen Peroxide Scavenging Activity

**Figure 3:**
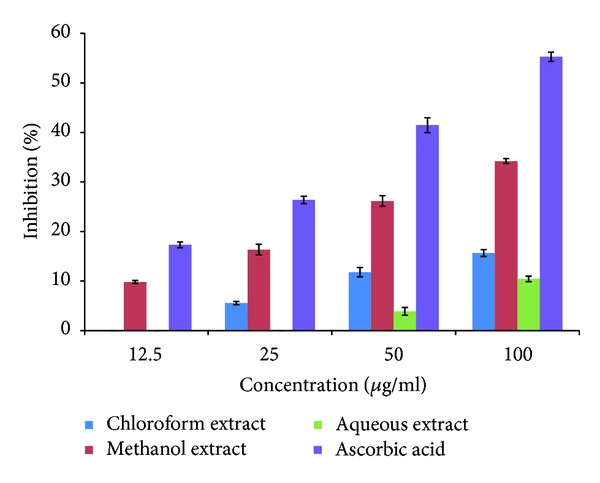
Inhibition of hydrogen peroxide radicals. The data represent the percentage of hydrogen peroxide radical inhibition (mean ± SEM) and experiments were performed in triplicate.

In this study, CNC, CNM, and CNA showed relatively poor hydrogen peroxide scavenging activities, albeit CNM being the most effective hydrogen peroxide scavenger which showed ~34% scavenging activity at 100 *μ*g/mL (Figure 3).

### 3.5. Antiproliferative Effect of *C. nutans* Extracts on the Growth of Tumor Cells

The antiproliferative activity of the *C. nutans* extracts against human liver hepatocellular carcinoma (HepG2), human neuroblastoma cell line (IMR-32), human lung cancer cell line (NCI-H23), human gastric cancer cell line (SNU-1), human colon adenocarcinoma cell line (LS-174T), human erythroleukemia cell line (K-562), human cervical cancer cell line (HeLa), and human Burkitt's lymphoma cell line (Raji) was shown in Table 1. Significant inhibition of cell proliferation was shown in HeLa and K-562 cells after treated with the *C. nutans* extract in aqueous at 100 *μ*g/mL (36.31 ± 1.52% and 40.94 ± 0.19%, resp.), but not the others. Methanol extract of *C. nutans* showed no activity towards four cancer cell lines, namely, the NCI-23, HeLa, K-562, and Raji cell lines. CNM showed relatively weak antiproliferative activity in IMR32, SNU-1, and LS-174T cell lines, although 41.88 ± 2.81% inhibitions were observed in HepG2 cell lines at 100 *μ*g/mL exhibited. CNC was the most potent antiproliferative agent against most cancer cell lines tested in this experiment. CNC showed the highest antiproliferative activity on K-562 and Raji cancer cell lines at 100 *μ*g/mL with percentage inhibition of 91.28 ± 0.03% (IC_50_ = 47.70 *μ*g/mL) and 88.97 ± 1.07% (IC_50_ = 47.31 *μ*g/mL), respectively. However, no effect was observed in IMR 32 cell lines.

To test whether the extract is cytotoxic to normal cells, human umbilical vein endothelial cells (HUVECs) were treated with all CNC, CNM, and CNA at the highest test concentration, which is 100 *μ*g/mL. The percentage inhibition was significantly lower in HUVECs compared to the other cancer cells (*P* < 0.05) (Table 1).

### 3.6. GC-MS Analysis of Chloroform Extract

**Figure 4:**
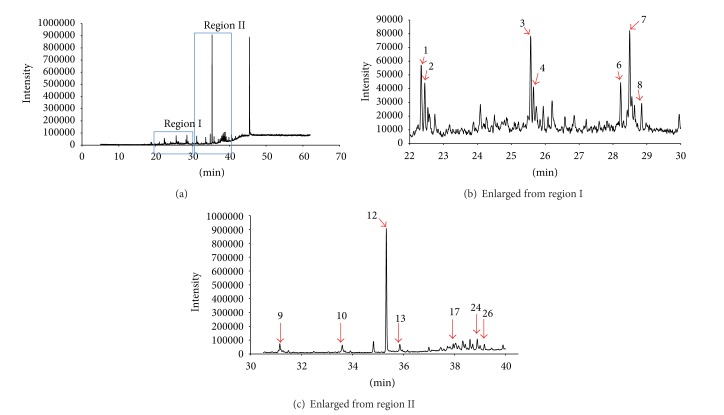
((a), (b), and (c)) Phytochemical constituents detected using GC-MS, with relative retention time (^*t*^ *R*) from chloroform extract of *C. nutans*, (b) Enlarged from region I, and (c) Enlarged from region II.

A GC-MS analysis was performed on chloroform extract of *C. nutans,* which exhibited relatively higher activity in antioxidant and anti-proliferative compared to the extracts in methanol and aqueous (Figure 4). Fourteen chemical constituents were identified, and the major chemical constituent was 1, 2-benzenedicarboxylic acid, mono (2-ethylhexyl) ester with relative peak area of 28.60% (Table 2). Previous reports suggested that 1, 2-benzenedicarboxylic acid, mono (2-ethylhexyl) ester has anti-microbial activity [20]. These phytochemical may also contribute to the medicinal activity, including antioxidant and antiproliferative properties.

## 4. Discussion

Past studies have shown that imbalance cellular redox homeostasis with elevated free radical production is one of the common causes of cancer initiation and progression and many other diseases [21]. Increased free radicals, the reactive oxygen species and reactive nitrogen species, in cancer cells correlate with the rapid progression to tumour development and promote metastasis, which often results in poor prognosis. The current treatment for cancer patients, such as chemotherapy and radiotherapy, may also contribute to oxidative damage to the tumour cells as well as the adjacent healthy cells. Many clinical trials have also suggested that antioxidants can shield the healthy cells from oxidative stress induced by these therapies, minimizing the subsequent side effects in cancer patients [22–24]. Thus, scavenging the free radicals with antioxidant may prove an alternate strategy to protect normal healthy cells from DNA damage and limit the progression of cancer cells. Nevertheless, accumulating evidence has also suggested that adaptation to extreme oxidative stress could occur in some transformed cancer cells [25]. These cancer cells could develop antioxidant defense system with enhanced scavenging capacity and survive intrinsic ROS toxicity through upregulation of survival proteins. For instance, *H-ras*-transformed cells with increased superoxide and hydrogen peroxide levels were noted with significant upregulation of antioxidant enzymes peroxiredoxin-3 and thioredoxin peroxidase [26]. This effect is likely to enable cancer cells to evade ROS mediated apoptosis. Therefore, it is possible that scavenging the radicals with exogenous antioxidant alone may not be effective to eliminate the transformed cancer. Furthermore, elevated ROS level in cancer cells were found to affect neighboring cells to acquire uncontrolled ROS production [27], through intercellular transfer of hydrogen peroxide (H_2_O_2_). Hence, antioxidant is still needed to stop ROS from spreading, and its role is more likely to salvage the unaffected cells adjacent to transformed cancer cells and limit tumour expansion.

Therefore, the concept of multitargeted therapy is introduced to exploit the use of single regimen to exert complex synergistic and antagonistic effects to act on multiple pathophysiological pathways in order to achieve optimal therapeutic outcome. *Clinacanthus nutans* (CN) leaves have previously been known to have anti-inflammatory [28] and antiviral properties [29, 30]. Recent report from Pannangpetch et al. [31] also documented that ethanolic extract of *C. nutans* leaves exhibited antioxidant activity and protective effect against oxidative hemolysis; however, the study had not been extended to investigate its antitumour properties. Here, we examined the effects of CN extracted in three different solvents: chloroform, methanol, and water. We observed that CN contained antioxidant elements and was capable of negating free radicals, after tested on diphenyl-1-picrylhydrazyl (DPPH), galvinoxyl radical, nitric oxide, and hydrogen peroxide scavenging assays, albeit with different CN extracts. However, results from DPPH were contradicting the data from nitric oxide scavenging assay. This may attribute to the solubility of the extracts in different testing system and the stereoselectivity of the radicals may also contribute to different antioxidant activity of the extract [32].

Emerging lay testimonies from cancer patients claimed that the herbs could eliminate the disease, suggesting their potential use for anticancer treatment. Our experiments proved that CN, particularly the extracts in chloroform, is capable of inhibiting cell proliferation of seven tested cancer cell lines, namely, the HepG2, IMR32, NCL-H23, SNU-1, HeLa, LS-174T, K562, and Raji cells. Although constituents in CNC failed to suppress the proliferation of IMR32 cancer cells, the inhibitory benefits could still be observed using extracts in methanol and in water. In CNC treated K562 cells and Raji cells, the IC_50_ values were 47.70 *μ*g/mL and 47.31 *μ*g/mL, which fall above the recommended IC_50_ value by National Cancer Institute (NCI) for crude extract, which is <20 *μ*g/mL. Although this data suggests that CNC may not be a strong anticancer regimen, the antioxidant and cancer inhibitory properties demonstrated in this experiment may still support the use of CNC as an alternate adjunctive therapy for cancer prevention or treatment. GC-MS analysis presented the volatile components of CNC, showing 14 phytochemicals at various concentrations, of which 1,2-benzenedicarboxylic acid, mono (2-ethylhexyl) ester being the most abundant in the extract, a common plasticizer which was found to possess antimicrobial activity [33]. Further studies are still needed to unveil the underlying mechanism and specific bioactive compounds phytochemical responsible for the observed activities.

## 5. Conclusion

In conclusion, chloroform extract of *C. nutans* leaves contains the most potent constituents capable of scavenging free radical and inhibiting the growth of cultured cancer cell lines. This observation suggests that phytochemical constituents present in chloroform extract could be used as an alternate adjunctive or chemopreventive regimen for patients at risk of cancers. However, further investigation study to understand the underlying mechanism and *in vivo* testing of the observed antitumor activity are needed to unveil its potential use in cancer therapy.

## Figures and Tables

**Table 1:** Anti-proliferative effect of chloroform, methanol and aqueous extract of *C. nutans* at various concentrations (3.125–100 *µ*g/mL) on eight different types of tumorigenic cell lines and normal cell, human umbilical veins endothelial cells (HUVECs). The growth inhibitory activity was normalized to control and expressed in percentage (%). Data represented in mean ± SEM in triplicate.

	HepG2	IMR32	NCI-32	SNU-1	HeLa	LS-174T	K562	Raji	HUVECs
Chloroform extract (*µ*g/mL)									
3.125	16.65 ± 1.84*	NA	27.03 ± 0.62*	6.63 ± 2.65	4.59 ± 0.83	21.78 ± 1.25*	9.46 ± 1.09	5.59 ± 1.18	
6.25	21.78 ± 1.21*	NA	33.30 ± 1.18*	15.16 ± 0.41*	8.47 ± 1.17	27.06 ± 1.04*	7.89 ± 0.35	15.47 ± 0.49*	
12.5	26.62 ± 0.73*	NA	36.65 ± 1.38*	18.17 ± 0.41*	16.16 ± 0.35*	33.98 ± 1.15*	9.99 ± 1.09	24.53 ± 0.43*	
25	29.97 ± 0.79*	NA	39.14 ± 0.69*	25.05 ± 0.68*	22.25 ± 0.19*	36.52 ± 0.70*	32.23 ± 1.06*	38.01 ± 0.28*	
50	32.49 ± 0.10*	NA	42.08 ± 0.75*	27.98 ± 0.18*	38.30 ± 0.08*	39.15 ± 0.42*	66.75 ± 0.47*	56.77 ± 0.23*	
100	38.76 ± 1.72*	NA	55.82 ± 1.23*	31.25 ± 1.09*	56.73 ± 0.12*	41.97 ± 0.94*	91.28 ± 0.02*	88.97 ± 1.06*	17.39 ± 3.49*
Methanol extract (*µ*g/mL)									
3.125	14.90 ± 1.41*	NA	NA	NA	NA	NA	NA	NA	
6.25	22.16 ± 0.92*	NA	NA	NA	NA	1.17 ± 0.68	NA	NA	
12.5	25.00 ± 0.67*	NA	NA	3.10 ± 1.49	NA	13.21 ± 1.79*	NA	NA	
25	26.93 ± 0.52*	4.57 ± 2.41	NA	9.06 ± 1.24	NA	21.06 ± 0.25*	NA	NA	
50	32.89 ± 1.50*	9.35 ± 0.75	NA	14.03 ± 1.32*	NA	22.66 ± 0.89*	NA	NA	
100	41.88 ± 2.81*	22.08 ± 1.22*	NA	21.32 ± 1.43*	NA	30.33 ± 0.91*	NA	NA	23.75 ± 1.49*
Aqueous extract (*µ*g/mL)									
3.125	4.02 ± 2.22	NA	NA	8.16 ± 0.55	9.37 ± 1.69	NA	NA	NA	
6.25	9.03 ± 0.56	NA	NA	13.27 ± 0.24*	15.74 ± 1.06*	NA	10.89 ± 0.49*	NA	
12.5	14.89 ± 0.38*	NA	NA	16.56 ± 0.62*	19.56 ± 1.34*	NA	12.48 ± 0.33*	NA	
25	16.15 ± 0.62*	0.36 ± 0.39	NA	20.51 ± 0.58*	24.84 ± 1.08*	NA	15.39 ± 0.42*	NA	
50	20.37 ± 0.65*	13.80 ± 0.70*	NA	22.30 ± 1.00*	27.42 ± 0.97*	NA	25.61 ± 0.83*	NA	
100	27.42 ± 0.80*	23.22 ± 0.63*	NA	27.57 ± 1.66*	36.31 ± 1.51*	NA	40.94 ± 0.19*	NA	17.31 ± 0.84*

*P < 0.05, (NA: no activity).

**Table 2:** Phyto-constituents identified in the chloroform extract of the leaves of *C. nutans* by GC-MS.

Peak	Name of the compound	Peak area (%)	RT
1	n-Pentadecanol	1.26	22.338
2	Eicosane	0.75	22.441
3	1-Nonadecene	1.64	25.570
4	Heptadecane	0.54	25.652
6	Dibutylphthalate	0.71	28.226
7	n-Tetracosanol-1	1.72	28.493
8	Heneicosane	0.33	28.558
9	Behenic alcohol	1.56	31.156
10	1-Heptacosanol	1.33	33.602
12	1,2-Benzenedicarboxylic acid, mono(2-ethylhexyl) ester	28.60	35.328
13	Nonadecyl heptafluorobutyrate	1.25	35.857
17	Eicosayl trifluoroacetate	1.10	37.949
24	1,2-Benzenedicarcoxylic acid, dinonyl ester	2.59	38.890
26	Phthalic acid, dodecyl nonylester	1.00	39.170

RT: retention time.
